# Foods for Arctic Explorers

**Published:** 1893-06-17

**Authors:** 


### Foods for Arctic Explorers.

Adventurers in the North Seas will certainly to-day be infinitely better off in the matter of food supplies than has been the fate of any of their predecessors, and most assuredly will stand a much greater chance of overcoming those difficulties which have hitherto proved insurmountable. The Bovril Company have had the preparing of supplies for Dr. Nansen, and are now preparing those for Mr. F. G. Jackson, who is also about to start on an expedition in search of that "mathematical point," the North Pole. On Saturday last Lord Playfair and the directors of the Bovril Company issued invitations for an inspection of their huge premises in Bath Street, City Road, to see the special foods in course of preparation for Mr. Jackson's expedition, and to hear some of the particulars of that expedition from Mr. Jackson himself. The various preparations manufactured specially for the Arctic explorers were on view in one of the large top rooms of the warehouse. The concentrated bovril is made in cakes, of a brownish colour, varying in size, and in its composition there are both vegetable and animal constituents.

It has been the object of the company, and one to which much consideration has been devoted, to provide the largest possible quantity of nutritious ingredients in the smallest possible bulk, and in such a manner as to secure its being quickly and easily made ready for use. The food is a mixture of some of the best extracts of meat, with a certain powdered essence of beef, which is procured from Australia, and consists of nothing but the pure lean of the meat. Most of the concentrated food will be in this form, only varying in point of flavour, but the Bovril Company are responsible for many ingenious methods for preparing the nourishment which is to last Mr. Jackson and his party through the two or three years which their expedition will probably take. Different kinds of dried vegetables, chocolates containing lime juice, meat jellies, &c., will go with the supply. On Saturday, after the visitors had seen something of the methods and principles on which bovril is made, everyone—the guests and many of the employés—gathered in one of the large rooms and listened to a few words from Lord Playfair, who spoke of the great increase in the scientific knowledge of the constituents of food, which was resulting in the production of such highly nutritious extracts of meat as those now being inspected. Lord Playfair then introduced Mr. Jackson, who gave a short description of his plans, and of the route he proposed to follow.

Sir Vesey Hamilton remarked upon the many advantages afforded to explorers now in comparison with their predecessors some forty years, and in the names of all present he wished Mr. Jackson every success in his enterprise, trusting that for the glory of England he might be beforehand with Dr. Nansen, though he too had the hearty good wishes of all who were interested in Arctic explorations. Adjournment was then made to another room, where tea, strawberries and cream, &c., awaited the visitors. In making a further inspection of the rooms where the preparation of the bovril, best known to the public in the little flat bottles is carried on, we were much impressed by the order and method everywhere reigning, and by the scrupulous cleanliness, and also the generally healthy and happy appearance of the employés, many of these being women, all wearing a uniform of blue dresses, white aprons, and blue and white caps. A little conversation with one of the manageresses increased this impression, and we left the premises of the Bovril Company feeling that for the future we should look upon the aforesaid little bottles with considerably increased respect.

